# Angiopoietin-like 4-Induced 3D Capillary Morphogenesis Correlates to Stabilization of Endothelial Adherens Junctions and Restriction of VEGF-Induced Sprouting

**DOI:** 10.3390/biomedicines10020206

**Published:** 2022-01-18

**Authors:** Athanasia Liabotis, Corinne Ardidie-Robouant, Philippe Mailly, Samaher Besbes, Charly Gutierrez, Yoann Atlas, Laurent Muller, Stéphane Germain, Catherine Monnot

**Affiliations:** 1 Center for Interdisciplinary Research in Biology (CIRB), College de France, CNRS, INSERM, Université PSL, F-75005 Paris, France; a.liabotisf@gmail.com (A.L.); corinne.ardidie@college-de-france.fr (C.A.-R.); philippe.mailly@college-de-france.fr (P.M.); samaher.besbes@gmail.com (S.B.); charly_gutierrez@ymail.com (C.G.); yoann.atlas@free.fr (Y.A.); laurent.muller@college-de-france.fr (L.M.); 2 Collège Doctoral, Sorbonne Université, F-75006 Paris, France

**Keywords:** angiopoietin-like 4, vascular endothelial growth factor, angiogenesis, adherens junction, in vitro 3D model, vascularization

## Abstract

Angiopoietin-like 4 (ANGPTL4) is a target of hypoxia that accumulates in the endothelial extracellular matrix. While ANGPTL4 is known to regulate angiogenesis and vascular permeability, its context-dependent role related to vascular endothelial growth factor (VEGF) has been suggested in capillary morphogenesis. We here thus develop in vitro 3D models coupled to imaging and morphometric analysis of capillaries to decipher ANGPTL4 functions either alone or in the presence of VEGF. ANGPTL4 induces the formation of barely branched and thin endothelial capillaries that display linear adherens junctions. However, ANGPTL4 counteracts VEGF-induced formation of abundant ramified capillaries presenting cell–cell junctions characterized by VE-cadherin containing reticular plaques and serrated structures. We further deciphered the early angiogenesis steps regulated by ANGPTL4. During the initial activation of endothelial cells, ANGPTL4 alone induces cell shape changes but limits the VEGF-induced cell elongation and unjamming. In the growing sprout, ANGPTL4 maintains cohesive VE-cadherin pattern and sustains moderate 3D cell migration but restricts VEGF-induced endothelium remodeling and cell migration. This effect is mediated by differential short- and long-term regulation of P-Y1175-VEGFR2 and ERK1-2 signaling by ANGPTL4. Our in vitro 3D models thus provide the first evidence that ANGPTL4 induces a specific capillary morphogenesis but also overcomes VEGF effect.

## 1. Introduction

Angiogenesis orchestrates the establishment of a functional vascular plexus from pre-existing vessels and therefore responds to oxygen and nutrients requirement during physiological or pathophysiological tissue remodeling. Sprouting angiogenesis consists of many stages regulated by a spatial and dynamic balance involving a multitude of actors. The development of in vitro settings recapitulating the complexity of the angiogenic process, capillary network morphogenesis and 3D spatial constraint remains a significant challenge for investigating therapeutic targets and tissue engineering [1]. Hypoxia is a major stimulus of angiogenesis, triggering the expression of growth factors like vascular endothelial growth factor (VEGF) as well as components or proteins associated to the extracellular matrix (ECM) [2,3]. Indeed, ECM-associated proteins are known to regulate angiogenesis both directly and through fine-tuning of VEGF signaling. In keeping with the latter, we previously reported that transglutaminase 2 is accumulated in the hypoxic endothelial ECM and limits VEGF_165_-induced angiogenesis through interaction with heparan sulfate proteoglycans [4].

Our team identified angiopoietin-like 4 (ANGPTL4), a member of the angiopoietin family as a target of hypoxia in endothelial cells and in human ischemic tissues [5]. ANGPTL4 is composed of an amino-terminal signal sequence, a coiled-coil domain, and a carboxy-terminal fibrinogen-like domain (FLD-ANGPTL4). ANGPTL4 is a secreted glycoprotein both released in the circulation and accumulated in the endothelial ECM through interactions with heparan sulfate proteoglycans [6,7]. ANGPTL4 displays multiple complex and debated functions. It regulates lipid metabolism by inhibiting the lipoprotein lipase (LPL) dimers present at the luminal surface of the capillary endothelium [8] through diverging mechanisms: ANGPTL4 inhibits LPL either by dissociating the active dimers into inactive monomers [9], or by unfolding the LPL monomers [10]. ANGPTL4 also acts on vascular processes, i.e., angiogenesis and permeability. As angiopoietin-2, the landmark family member, ANGPTL4 is a context-dependent modulator of vascular permeability [11,12] and maintains vascular integrity in retinopathy, myocardial infarction, and stroke [13,14,15]. We demonstrated that the vasoprotective effect of ANGPTL4 is mediated by its interaction with α_v_β_3_ integrin at the endothelial cell surface leading to Src recruitment and sequestration away from VEGFR2 [16]. Assessing how ANGPTL4 regulates the dynamic patterning of VE-cadherin in the sprouting capillaries is essential to understand its involvement in angiogenesis. Indeed, during angiogenesis, regulation of the VE-cadherin patterns is characterized by iterative changes between serrated structures and reticular plaques in the dynamic areas whereas linear structures remain in the stable junctions [17,18,19,20]. Cell migration in a capillary sprout is associated with remodeling of the endothelial adherens junctions and with subsequent formation of new overlapping reticular plaques [18,20]. In this study, we therefore aimed to decipher the role of ANGPTL4 on angiogenesis, capillary architecture, and adherens junction patterning in a 3D context. Furthermore, assessing ANGPTL4 involvement on VEGF-induced capillary formation and integrity is of major interest, since VEGF is expressed concomitantly with ANGPTL4 in hypoxia. One of the first angiogenic steps consisting in the jamming to unjamming transition of endothelial monolayer, associated with an anisotropic cell distribution leads to the fluid-like state of the cell population as previously reported for VEGF [21]. We demonstrate here for the first time that ANGPTL4 limits the VEGF-induced jamming to unjamming transition.

Altogether, we demonstrate that ANGPTL4 induces barely branched and thin endothelial capillaries and limits VEGF-induced sprouting and capillary morphogenesis using relevant in vitro 3D capillary models and purified human recombinant ANGPTL4. These effects are associated with the restriction of endothelial cell elongation, the reduction in endothelial cell migration, and the preservation of thin and linear cell–cell adherens junctions. The regulatory role of ANGPTL4 is mediated by short- and long-term regulation of P-Y1175-VEGFR2/ERK1-2 signaling. These studies highlight the regulatory impact of ANGPTL4 on the angiogenic process mediated by the restriction of the endothelial cell–cell junction patterning.

## 2. Materials and Methods

### 2.1. Cell Culture, Antibodies, and Reagents

Human umbilical vein endothelial cells (HUVEC) were prepared and grown as previously described [22] from human umbilical cords provided by AP-HP, Hôpital Saint-Louis, Unité de Thérapie Cellulaire, CRB-Banque de Sang de Cordon, Paris, France (authorization number AC-2016-2759). Normal human dermal fibroblasts (NHDF) were from PromoCell GmbH (Heidelberg, Germany). Experiments were performed between passages 1 and 5 for each primary cell culture. Polyclonal anti-VEGFR2 and anti-phospho-VEGFR2 (P-Y951 or P-Y1175) antibodies and monoclonal anti-phospho-ERK1/2 (P-T202/P-Y204) and β-catenin antibodies were from Cell Signaling Technology (Boston, MA, USA). Polyclonal anti-ERK1/2, thrombin, and fibrinogen were from Merck KGaA (Darmstadt, Germany). Polyclonal anti-beta actin was from Abcam (Cambridge, MA, USA). Alexa Fluor™488 monoclonal VE-cadherin antibody, Alexa Fluor™488, and BODIPY™ 558/568 Phalloidin were from ThermoFisher Scientific (Waltham, MA, USA). Human VEGF_165_ (called VEGF) was from R&D Systems (Minneapolis, MN, USA). Human full-length ANGPTL4 and FLD-ANGPTL4 (extending from residues 171 to 406) were produced and purified as previously described [7]. Endothelial cell basal (ECBM2), growth (ECGM2), and fibroblast growth (FGM2) media were from PromoCell GmbH. NHDF-conditioned medium was obtained from confluent culture in ECGM2 and collected every day between 2 and 10 days.

### 2.2. Three-Dimensional Angiogenesis Model 

#### 2.2.1. Capillary Formation and Staining

Three-dimensional endothelial sprouting assay was performed in CellCarrier-96 Ultra microplates (PerkinElmer, Waltham, MA, USA) as modified from [22]. Cytodex^®^3 beads (Merck KGaA) were stained by Alexa Fluor™568 carboxylic acid, succinimidyl ester (Molecular Probes, Eugene, OR, USA). Capillaries grew for 4 days in absence (control condition) or presence of VEGF (2.5 ng/mL) and/or full-length ANGPTL4 (2.5 µg/mL) or FLD-ANGPTL4 (4 µg/mL) (in respect to the same concentration taking account to respective molecular weight) added to the NHDF-conditioned medium. Media were changed daily. Gels were fixed in 4% PFA for 30 min and permeabilized using 0.5% TritonX-100 for 30 min. Alexa Fluor™-conjugated VE-cadherin antibody or -Phalloidin were incubated in 0.1% TritonX-100 at 4 °C overnight. DAPI staining was performed for 15 min at room temperature.

#### 2.2.2. Morphometric Analysis of Capillaries

Images were acquired using a Zeiss Axiozoom Apotome 2 microscope (Zeiss, Oberkochen, Germany) with a 2.3× objective at 1.6× zoom for the automatic fast-acquisitions of the fluorescent beads and at 10× zoom for the 3D reconstruction of the capillary network sprouting from the beads. An in-house ImageJ (Rasband, W.S., ImageJ, U. S. NIH, Bethesda, MD, USA) plugin was developed to perform morphometric analysis of capillaries by quantifying number, total branch number, total length of capillaries per bead as well as capillary diameter. Briefly, fluorescent beads (DSRed channel) were automatically detected in each well of the 96-well plate using an in-house Python script, driving microscope. After segmentation, bead center and diameter were computed using ImageJ Macro. A corresponding 3D sphere displaying a 25 µm-increased radius was drawn in GFP Channel with each voxel set at zero to remove cells in contact with the bead. 3D vessel network (GFP channel) was segmented and skeletonized, quantification of capillaries was performed using Analyze Skeleton and Sholl libraries. For qualitative analysis of VE-cadherin patterns, images were acquired using a Leica confocal SP-5 inverted microscope at 63× magnification (Leica Microsystems Heidelberg, Germany). 3D reconstruction images and video were performed using Imaris software (Bitplane, Belfast, UK).

### 2.3. Three-Dimensional Models of Endothelial Cell Behavior 

#### 2.3.1. Constraining Model for Cell Shape

Endothelial cells seeded on beads were transferred after 24-h culture into µ-Slide 8 uncoated wells (Ibidi, Martinsried, Germany) and further cultured for 5 h in absence (control condition) or presence of VEGF (50 ng/mL) and/or ANGPTL4 (2.5 µg/mL) added to ECBM2. Fixation, permeabilization, and F-actin, nucleus or VE-cadherin staining were performed as described in Section 2.2.1. Images were acquired using Leica confocal SP5-inverted microscope (63× objective/1.4 NA). Cell perimeter and circularity were quantified using ImageJ.

#### 2.3.2. Spheroid Assay for Cell-Cell Junction Integrity

Endothelial spheroids (1000 cells) were performed as previously described [6] and transferred into Ibidi µ-Slide 8 uncoated wells. Spheroids were cultured for 24 h in absence (control condition) or presence of VEGF (50 ng/mL) and/or ANGPTL4 (2.5 µg/mL) added to ECGM2. Spheroids were fixed in 4% PFA for 30 min and embedded in 1% low melting agarose. Permeabilization and nucleus or VE-cadherin staining were performed as described in Section 2.2.1. Images were acquired using a Leica confocal SP5-inverted microscope (63× objective/1.4 NA). Spheroid circularity index was quantified using ImageJ.

#### 2.3.3. Model of 3D Migration

Endothelial cells seeded on beads were embedded after 24-h culture into a 2.5 mg/mL fibrin gel and further cultured for 48 h in absence (control condition) or presence of VEGF (2.5 ng/mL) and/or ANGPTL4 (2.5 µg/mL) added to ECGM2. Fixation, permeabilization, and F-actin or nucleus staining were performed as described in Section 2.2.1. Images were acquired using Zeiss Observer Apotome 2 (10× objective). Three-dimensional migration was assessed using Imaris software for both quantification of the maximal distance covered by the cells from the bead (over the 10% maximal values of nucleus distances) and of the nuclei number emerging out of the bead surface.

### 2.4. Protein Extraction and Immunoblotting

Confluent endothelial cells were grown on Petri dish for 3 days in ECGM2. Cells were depleted for 5 h in ECBM2 1% BSA before short-term (2 min) stimulation. For short- and long-term (5 h) stimulation, cells were cultured for 24 h in absence (control condition) or presence of VEGF (50 ng/mL) and/or ANGPTL4 (2.5 or 5 µg/mL) added to ECBM2 1% BSA. Cell lysates were collected and analyzed as previously described [4].

### 2.5. Statistical Analysis

Statistical analyses were performed with Kruskal Wallis or one-way ANOVA tests according to variances, sample sizes, and distributions. Statistics were computed in GraphPad PRISM 6 software (GraphPad Software Inc., San Diego, CA, USA). The specific statistical test performed for each experiment is included in the appropriate figure legend.

## 3. Results

### 3.1. ANGPTL4 Induces Specific 3D Capillary Morphogenesis and Overcomes VEGF Effect

**Figure 1:**
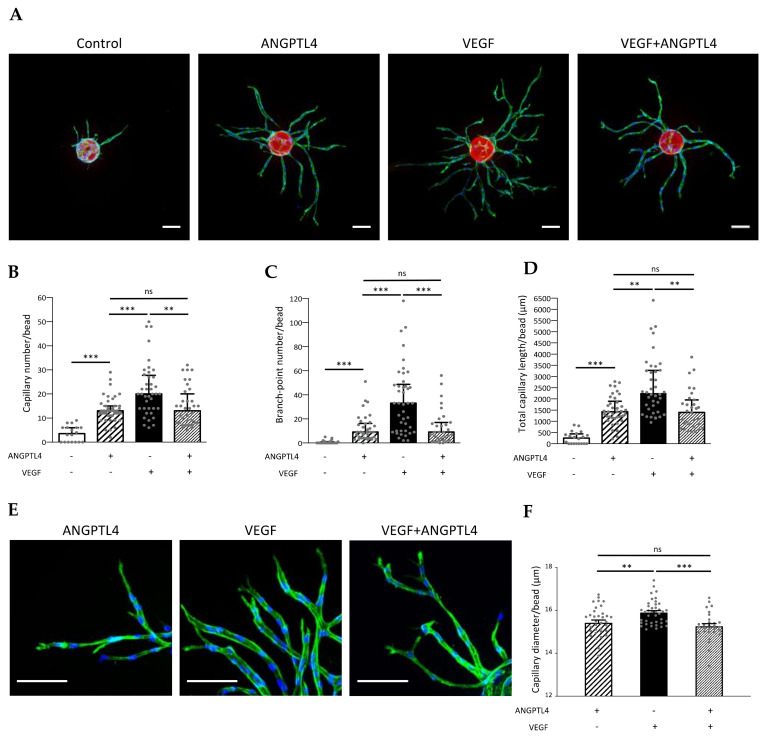
Regulation of 3D capillary morphogenesis by ANGPTL4. (**A**) 3D capillary formation was assessed after 4 days of culture in absence (control) or presence of ANGPTL4 (2.5 µg/mL) and/or VEGF (2.5 ng/mL). Capillaries sprouting from fluorescent bead (red) were stained for F-actin (green) and nuclei (blue). Scale bar: 100 µm. (**B**) Median values of capillary number/bead (+interquartile). (**C**) Median values of branch-point number/bead (+interquartile). (**D**) Median values of total capillary length/bead (+interquartile). (**E**) Capillaries stained for F-actin (green) and nuclei (blue). Scale bar: 100 µm. (**F**) Mean values of capillary diameter/bead (+SEM). Values were measured in three independent experiments of at least triplicate wells. ns: *p* > 0.05, ** *p ≤* 0.01, *** *p ≤* 0.001 ((**B**–**D**): Kruskal–Wallis, (**F**): one-way ANOVA).

Regulation of capillary formation and morphogenesis by ANGPTL4 was explored in presence or absence of VEGF using in vitro 3D angiogenesis models characterized by lumen formation and deposition of a polarized basement membrane [4,22]. Endothelial cells (HUVEC) were seeded on fluorescent beads, embedded in fibrin gel and cultured in presence of conditioned medium of normal human dermal fibroblasts (NHDF). Quantitative analyses of capillaries were performed from acquisitions (Video S1 for 3D reconstruction from Z-stack images) sequentially processed into 3D mask and 3D morphometric image (Supplementary Figure S1). In control conditions, capillaries barely sprouted from the fluorescent beads. As expected, VEGF induced the formation of a dense network of ramified capillaries. Whereas ANGPTL4 alone induced capillary formation, it limited sprouting and branching processes induced by VEGF (Figure 1A). Morphometric analysis demonstrates that capillary number (Figure 1B), branch-point number (Figure 1C), and total capillary length per bead (Figure 1D) were significantly increased by ANGPTL4 when compared to control, and reached similar values in presence or absence of VEGF. Detailed analysis of capillary length including the main and the related branches reveals that whereas the small capillaries (shorter than 50 µm) were the most abundant in control condition, ANGPTL4 and/or VEGF treatments induced a shift toward longer capillaries, extending from 50 to 200 µm (Supplementary Figure S2A). ANGPTL4-induced capillaries were similarly abundant in each length range in presence or absence of VEGF, but less abundant than the VEGF-induced capillaries in each corresponding range. However, percentages of capillaries per length range were similarly distributed in these three conditions and distinct from the control condition (Supplementary Figure S2B). These data demonstrate that ANGPTL4 alone or in presence of VEGF induced the formation of short and long barely branched capillaries whereas VEGF alone induced short and long ramified capillaries (Figure 1C). ANGPTL4 effect on capillary morphogenesis was further assessed on the capillary mean diameter (Figure 1E). Capillaries displayed smaller diameters in presence of ANGPTL4 alone (15.42 ± 0.12 µm) or combined with VEGF (15.26 ± 0.12 µm) than in presence of VEGF alone (15.90 ± 0.08 µm) (Figure 1F). These results suggest that ANGPTL4 induces a specific capillary morphogenesis that is dominant over VEGF-induced angiogenesis.

Since human ANGPTL4 is present in the secretion medium of endothelial cells and circulates in human serum [23,24] where it can be cleaved thereby generating a circulating carboxyl-terminal fibrinogen-like domain, FLD-ANGPTL4 [25,26], we assessed the role of FLD-ANGPTL4 on 3D capillary formation. FLD-ANGPTL4 alone stimulated angiogenesis more efficiently than full-length ANGPTL4 and reduced VEGF-induced angiogenesis although to a lesser extent than full-length ANGPTL4 (Supplementary Figure S3). These results demonstrate that the FLD-ANGPTL4 partially recapitulates the dominant effect of the full-length ANGPTL4 over the VEGF-induced capillary morphogenesis.

### 3.2. ANGPTL4 Regulates VE-Cadherin Patterning in the Sprouting Capillaries

**Figure 2:**
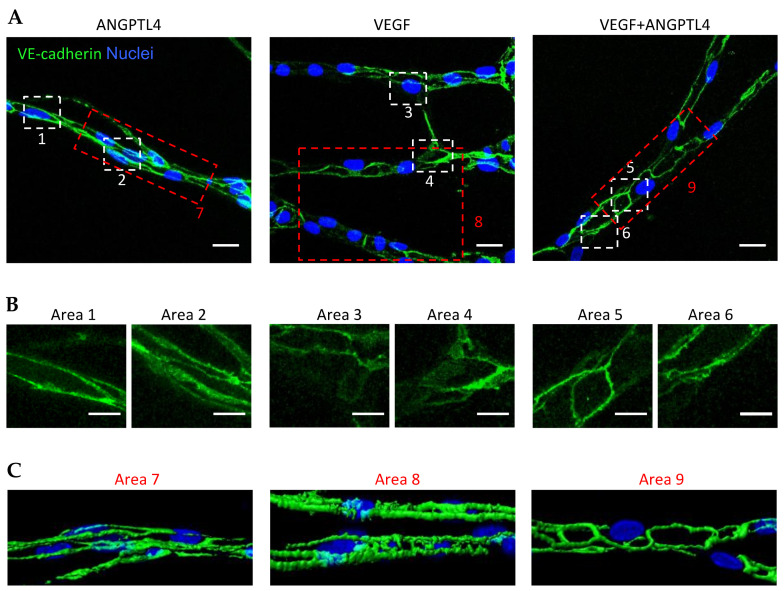
Regulation of VE-cadherin pattern in sprouting capillaries by ANGPTL4. (**A**) Z-stack projection of sprouting capillaries after 4 days of culture with ANGPTL4 (2.5 µg/mL) and/or VEGF (2.5 ng/mL). Capillaries were stained for VE-cadherin (green) and nuclei (blue). Scale bar: 20 µm. White boxed areas are presented in (**B**). Scale bar: 10 µm. (**C**) 3D reconstruction of capillaries presented in red boxed images. These images are representative of three independent experiments.

The present findings suggest that ANGPTL4 displays VEGF-dependent activity reminiscent of the outcome of Ang-2 in the presence or absence of VEGF-A [27]. As shown previously, VE-cadherin dynamics control VEGF-induced signal transduction and is therefore essential for the regulation of angiogenesis [28,29]. We thus analyzed VE-cadherin patterns in sprouting capillaries in the 3D angiogenesis model (Figure 2A). Depending on the treatment, VE-cadherin organization displayed a range of patterns from linear and continuous structures revealing straight cell–cell junctions (as example Area 1 in Figure 2B) to reticular plaques covering large cell–cell contact surface (as example Area 4 in Figure 2B). ANGPTL4-induced capillaries displayed linear or small reticular patterns of VE-cadherin mainly at lateral junctions (Figure 2B, Areas 1 and 2, respectively) whereas VEGF-induced capillaries contained VE-cadherin included in serrated structures or in large reticular plaques (Figure 2B, Areas 3 and 4, respectively). In capillaries grown in presence of ANGPTL4 and VEGF, VE-cadherin organization displayed linear pattern or small reticular structures (Figure 2B, Areas 5 and 6, respectively). Three-dimensional image reconstruction of the VE-cadherin patterns further confirms thin and linear structure in ANGPTL4-induced capillaries in absence or presence of VEGF (Figure 2C Areas 7 and 9, respectively) and serrated or thick structures in VEGF-induced capillaries (Figure 2C, Area 8).

ANGPTL4 therefore stabilizes VE-cadherin-containing junctions in sprouting capillaries by promoting linear or small reticular patterns and counteracts the formation of the large reticular VE-cadherin plaques and serrated structures promoted by VEGF. ANGPTL4 thus counteracts VEGF-induced sprouting and branching processes thereby reducing both capillary and branchpoint numbers when compared to VEGF.

### 3.3. ANGPTL4 Regulates Endothelial Cell Shape and Junctions from the First Angiogenic Step

**Figure 3:**
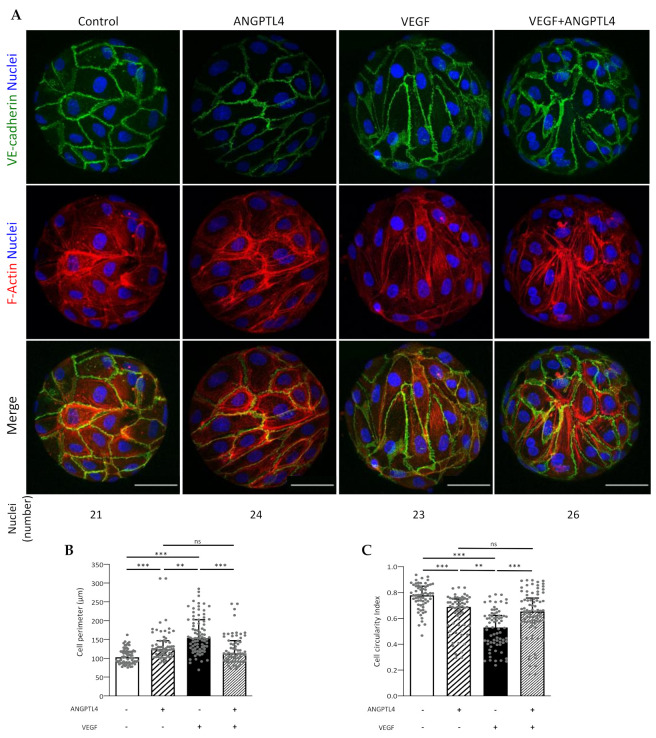
Regulation of migratory-related endothelial behavior by ANGPTL4 and VEGF in the early step of angiogenesis. (**A**) Endothelial cells seeded on beads and incubated without or with ANGPTL4 (2.5 µg/mL) and/or VEGF (50 ng/mL) for 5 h. Cells were fixed and labelled for VE-cadherin (green), F-actin (red), and nuclei (blue). The number of visible nuclei per bead is indicated below the panels. Scale bar: 50 µm. (**B**) Median values of cell perimeter (+interquartile). (**C**) Median values of cell circularity index (+interquartile). Values were measured on 62 to 83 cells per conditions in at least three independent experiments. ns: *p* > 0.05, ** *p* ≤ 0.01, *** *p* ≤ 0.001 (Kruskal–Wallis).

Endothelial cell elongation is essential for triggering angiogenesis [29] and is responsible for VE-cadherin patterning along cell junctions [30]. We assessed the effect of ANGPTL4 on cell shape in the early angiogenic step, using the cell properties of curvotaxis [31]. Indeed, HUVEC, which have been previously characterized for their high sensitivity to curvature [32] were submitted to spatial mechanical constraints (approx. 700 nuclei per mm^2^ for each condition) when seeded on beads. As previously reported using various constraining substrates, endothelial cells were prone to plasticity and reorientation of their cell body leading to a homogenous migration pattern via a fluidization process [33], depending on the stimulus, whereas the total amount of VE-cadherin and β-catenin remained constant (Supplementary Figure S4). Untreated cells displayed isotropic orientation, polygonal shape associated with cortical F-actin (Figure 3A). VEGF-treated cells displayed anisotropic orientation, elongated shape associated with actin stress fibers. ANGPTL4, in presence or absence of VEGF induced an isotropic orientation with moderate cell elongation associated with cortical actin. Quantification of cell perimeter and circularity index compared to control condition (Figure 3B,C, respectively) demonstrates that VEGF strongly induced cell shape transition by increasing elongation whereas ANGPTL4 increased cell elongation to a lesser extent, whether in absence or presence of VEGF. 

**Figure 4:**
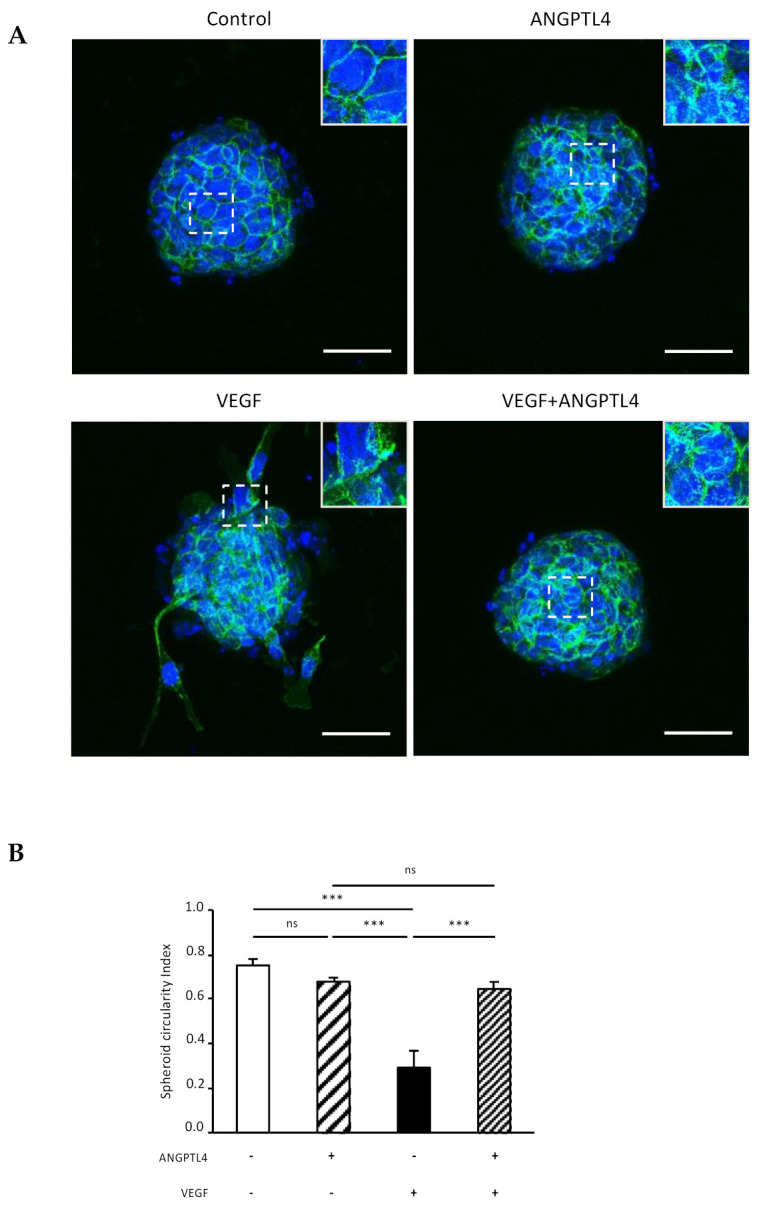
Regulation of VE-cadherin and endothelial spheroid integrity by ANGPTL4 and VEGF. (**A**) Endothelial spheroids treated without or with ANGPTL4 (2.5 µg/mL) and/or VEGF (50 ng/mL) for 24 h. Spheroids were fixed and labelled for VE-cadherin (green) and nuclei (blue). White boxed areas are presented in top right insert. Scale bar: 50 µm. (**B**) Mean values of spheroid circularity index (+SEM). Values were measured in three independent experiments. ns: *p* > 0.05, *** *p ≤* 0.001 (one-way ANOVA).

We next asked whether ANGPTL4 would protect VEGF-induced VE-cadherin destabilization at a later stage, using endothelial spheroids cultured in absence or presence of ANGPTL4 and/or VEGF (Figure 4A). In control condition, dense and unaltered spheroids mostly displayed thin and straight VE-cadherin pattern. ANGPTL4 treatment did not affect spheroid integrity when compared to control condition whereas VEGF treatment induced spheroid dissociation. When combined with VEGF, ANGPTL4 preserved spheroid integrity, as assessed by spheroid circularity index (Figure 4B). Indeed, only VEGF treatment led to a significantly decreased index. ANGPTL4-treated spheroids displayed in absence or presence of VEGF, heterogenous VE-cadherin patterns although containing smaller reticular structures compared to VEGF-treated spheroids. These results demonstrate that ANGPTL4 counteracts VEGF-induced destabilization of cell–cell junctions and therefore maintains cohesive contacts between endothelial cells.

These results suggest that ANGPTL4 counteracts the VEGF-induced individual cell elongation associated with the fluidization of endothelial cell population that occurs during the early steps of the sprouting process. ANGPTL4 then maintains the cohesive VE-cadherin pattern in lateral linear structures in the later steps of capillary morphogenesis, in a dominant manner over VEGF.

### 3.4. ANGPTL4 Regulates Endothelial Cell 3D Migration and VEGF/VEGFR2-Downstream Pathway 

**Figure 5:**
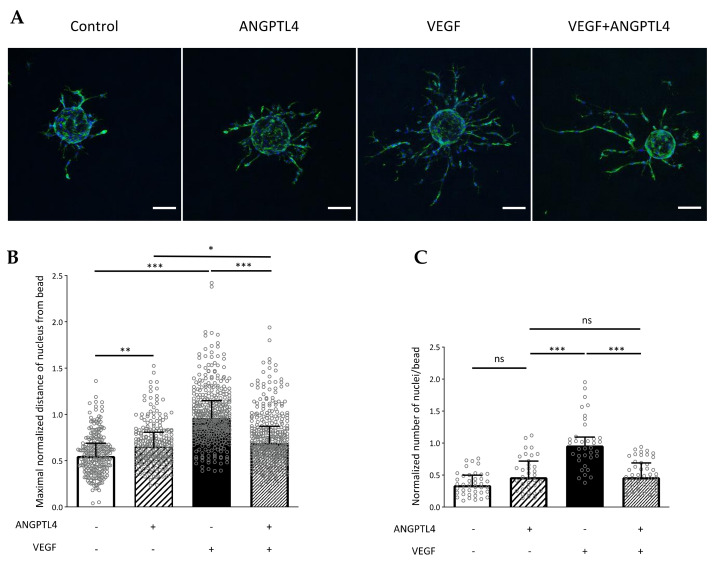
Regulation of 3D endothelial cell migration by ANGPTL4 and VEGF. (**A**) Cell responses were assessed by incubating endothelial cells seeded on beads with endothelial growth medium without or with ANGPTL4 (2.5 µg/mL) and/or VEGF (2.5 ng/mL) for 48 h. Cells were fixed and labelled for F-actin (green) and nuclei (blue). Scale bar: 100 µm. (**B**) Median values of normalized maximal distance covered by cell (nucleus) from the bead for each condition (+interquartile). (**C**) Median values of normalized number of nuclei emerging from a bead (+interquartile). Nuclei closely surrounding the bead are not considered. Individual values are normalized to the mean value of VEGF condition for each experiment. Values were measured in three independent experiments. ns: *p* > 0.05, * *p* ≤ 0.05, ** *p* ≤ 0.01, *** *p* ≤ 0.001 (Kruskal-Wallis).

VEGF-induced cell elongation and the subsequent VE-cadherin pattern are of major importance for polarized migration [18]. As demonstrated above, ANGPTL4 interfered with endothelial cell elongation and subsequent formation of large cell–cell contact surface containing reticular VE-cadherin structures induced by VEGF. We therefore assessed the regulation of 3D individual cell migration by ANGPTL4 using cell-seeded beads embedded in fibrin gel as in [4,22] (Figure 5A). Quantifying the maximal distance covered by cells from the bead reveals that individual cell migration was significantly increased by ANGPTL4 when compared to control condition, although to a lesser extent than VEGF (Figure 5B). Furthermore, ANGPTL4 strongly inhibited VEGF-stimulated migration. We also analyzed whether the overcoming effect of ANGPTL4 on VEGF occurred on cell proliferation (Figure 5C). Quantification of the total number of nuclei of cells emerging out from the bead reveals that ANGPTL4 did not stimulate cell proliferation whereas VEGF induced a two-fold increase in cell number when compared to control. In these conditions, ANGPTL4 also inhibited VEGF-induced proliferation.

**Figure 6:**
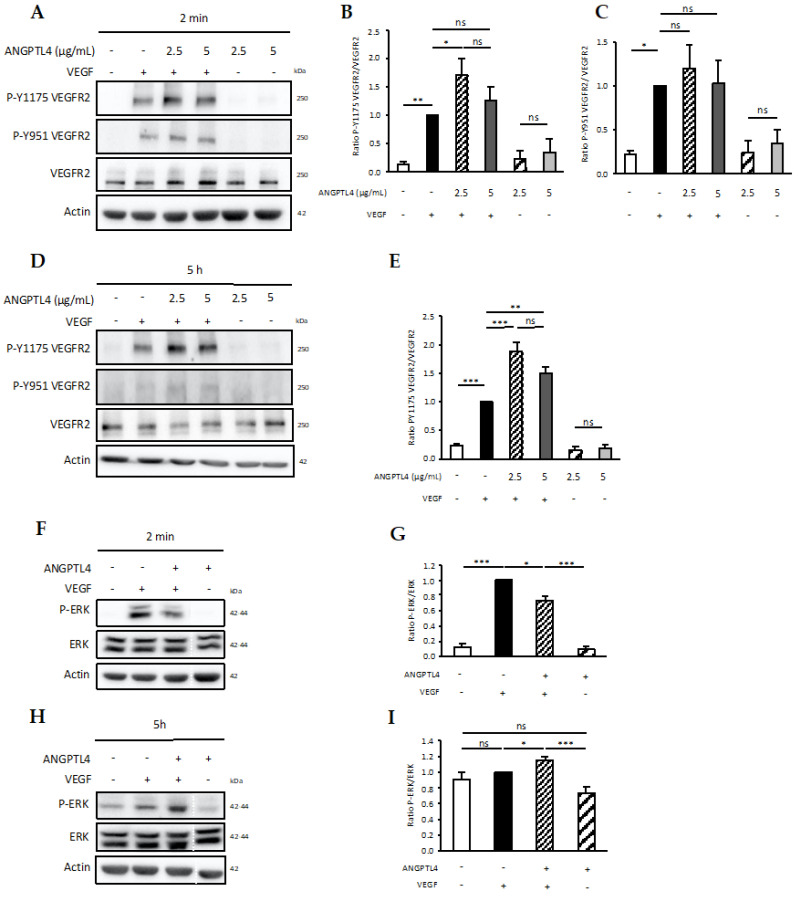
Short- and long-term regulation of VEGFR2 signaling. Endothelial cells were stimulated without or with ANGPTL4 (2.5 or 5 µg/mL) and/or VEGF (50 ng/mL). Phosphorylation of VEGFR2 at tyrosine sites 951 and 1175 was analyzed after 2 min (**A**–**C**) or 5 h (**D**,**E**) stimulation. Phosphorylation of ERK was analyzed after 2 min (**F**,**G**) or 5 h (**H**,**I**) treatment by ANGPTL4 (2.5 µg/mL) and/or VEGF (50 ng/mL). Immunoblots are representative of several independent experiments (3 for (**B**,**E**,**G**,**I**); 5 for (**C**)). Blot image cuts are indicated by dotted white lines. Actin immunoblot was used as loading control. Graphs represent mean values (+SEM) of the ratio of phosphorylated protein over total protein. Individual values are normalized to the mean value of VEGF condition for each experiment. ns: *p* > 0.05, * *p* ≤ 0.05, ** *p* ≤ 0.01, *** *p* ≤ 0.001 (one-way ANOVA).

These results showed that (i) dynamics of local VE-cadherin patterns are differentially regulated by ANGPTL4 and VEGF during the sprouting process; (ii) ANGPTL4 stimulates 3D migration of endothelial cells but counteracts VEGF-induced migration thus sustaining moderate migration. We next analyzed the signaling events that could mediate these effects. The VEGFR2/ERK1-2 pathway is involved in short and long-term regulation of migration by VEGF [34]. In response to short-term (2 min) VEGF treatment, Tyr951 and Tyr1175 were phosphorylated (P-Y951 and P-Y1175, respectively) (Figure 6A–C). Although ANGPTL4 alone did not stimulate VEGFR2 phosphorylation, it further increased VEGF-induced P-Y1175, but not P-Y951. Long-term (5 h) VEGF treatment induced a sustained phosphorylation of P-Y1175 (Figure 6D,E) whereas P-Y951 was completely abolished (Figure 6D). Interestingly, P-Y1175 was further increased by ANGPTL4 (Figure 6D,E). As P-Y1175 VEGFR2 is highly involved in the activation of ERK1-2 signaling [35], we further assessed VEGF-induced ERK1-2 phosphorylation (P-ERK) in absence or presence of ANGPTL4. Whereas P-ERK was stimulated by 2 min-VEGF treatment (Figure 6F,G), it was abolished at 5 h-treatment (Figure 6H,I). However, VEGF-induced P-ERK was significantly decreased at short-term and conversely stimulated at long-term in presence of ANGPTL4. These results emphasize distinct mechanisms involved in the time-dependent regulation by ANGPTL4 of the VEGF-induced VEGFR2 activity. Furthermore, the total amount of VEGFR2 remained unchanged whatever the experimental conditions of short-term stimulation (Supplementary Figure S5A). However, after long-term treatment, VEGF reduced the total amount of VEGFR2, whereas this effect was abolished by ANGPTL4 (Supplementary Figure S5B). 

Altogether, these data shed light on the role of ANGPTL4 on capillary morphogenesis and VE-cadherin patterning. ANGPTL4 induces thin capillary network, displaying linear adherens junctions and counteracts VEGF-induced dense and ramified network formation. ANGPTL4 induces a shift from long migrating cells with serrated junctional structures and actin fibers as induced by VEGF to polygonal cells with linear junctions and cortical actin, thereby regulating endothelial cell fluidization, migration, and long-term P-Y1175-VEGFR2/ERK1-2 signaling.

## 4. Discussion

Various important functions have been assigned to ANGPTL4. Whereas it is clearly involved in regulating lipid metabolism [36,37], its role in vascular biology has been a matter of debate for long. We and others have previously reported a role of ANGPTL4 in repressing angiogenesis and vascular permeability [11,13,14,38] and other studies reported that ANGPTL4 is an alternative angiogenic factor in the promotion of the retinal vascularization [39] and acts synergistically with VEGF to destabilize the vascular barrier, such as in the retina [12]. Using 3D models that faithfully recapitulate all the steps of the angiogenesis process, we demonstrate here that ANGPTL4 is promoting the formation of barely ramified and thin endothelial capillaries. In contrast, ANGPTL4 suppresses VEGF-induced capillary sprouting. This ANGPTL4-specific context-dependent response is mediated by the regulation of VE-cadherin patterning associated with the migratory capacities of the endothelial cells throughout the sprouting process. Indeed, ANGPTL4 counteracts the VEGF-induced unjamming of the endothelial cell population during the early step of the sprouting process and dominantly maintains the local VE-cadherin pattern in lateral linear structures in later steps of the capillary morphogenesis. Furthermore, ANGPTL4 has the ability to promote 3D endothelial cell migration but also to counteract that induced by VEGF. Mechanistically, this effect is mediated via differential short- and long-term regulation of P-Y1175-VEGFR2 and ERK1-2 signaling by ANGPTL4.

Angiogenesis and vascular permeability are two complex processes controlled by many microenvironmental cues, among them spatial constraints, ECM composition, and growth factor bioavailability [3]. In line with the consensus guidelines regarding the use of appropriate angiogenesis assays [40], we set up accurate 3D models, which maintain spatial and/or environmental constraints and integrate required cues for optimal angiogenesis studies. Indeed, confluent endothelial cells growing on 2D monolayer display high heterogeneity for both cell shape and adherens junction organization [17]. This heterogeneity is due to complex isotropic movements inside the cell population resulting from the multiplicity of uncoordinated individual migrations.

The 3D model of endothelial cell culture on beads mimics both the spatial constraints and the first angiogenic step, i.e., elongation and anisotropic orientation of the cells that then display homogenous shapes and VE-cadherin patterns in contrast to 2D monolayer. Previous studies have demonstrated that VEGF promotes the transition from jamming to unjamming pattern of endothelial monolayer that allows the dynamic formation of the endothelial sprout and an increase of the shape index (*p* = *P/*√*A*, where *P* and *A* are the cell perimeter and the projected area, respectively) [21]. Our data are in agreement with these results as we show that VEGF induces an increased cell perimeter, a decreased cell circularity index, and an anisotropic cell orientation when compared to control condition. Furthermore, we demonstrate for the first time that ANGPTL4 alone also increases cell elongation although to a lesser extent than VEGF and, in contrast, counteracts VEGF-induced cell elongation. Our study thus highlights that ANGPTL4 restricts the VEGF-induced fluidization process.

In addition, the later events such as regulation of adherens junction integrity were also analyzed using the 3D model of capillary sprouting. Endothelial capillaries are polarized, covered by a basement membrane and contain a lumen. Since ANGPTL4 interacts with α_v_β_3_ integrins and stabilizes the α_v_β_3_ integrin-VEGFR2 [16], analysis of these regulatory mechanisms also requires 3D model that involves both cell–matrix and cell–cell interactions. Thus, 2D models using simple layer of ECM components or extracts such as Matrigel display major limitations to properly decipher the role of ANGPTL4 on angiogenesis and cell–cell junction integrity. We here show that ANGPTL4 whether or not in the presence of VEGF, regulates endothelial cell adherens junctions in the sprouting capillaries by maintaining the VE-cadherin in thin linear structures and therefore limits the large reticular structures induced by VEGF. These results are in line with our previous in vivo data reporting larger diameter of capillaries and veins in *angptl4-*deficient mouse retina [13]. Regulation of capillary formation and integrity are indeed two associated processes as revealed by the dynamics of junction-associated structures and of VE-cadherin organization in a sprout. Large structures are mainly formed at the cell poles whereas small structures are present at the lateral junctions [18]. Altogether, our results strongly suggest that ANGPTL4 reduces the number of capillaries and branches by limiting the VEGF-induced fluidization of the endothelial population and by reducing the reticular junctions formed between actively migrating endothelial cells. In agreement with previous results [41], we here demonstrate that ANGPTL4 alone stimulates endothelial cell 3D migration without affecting proliferation. Conversely, ANGPTL4 reduces VEGF-induced migration and inhibits VEGF-induced proliferation. These data highlight distinct regulatory mechanisms of 3D migration and proliferation by ANGPTL4 in the presence or absence of VEGF, whereas the effect on preservation of endothelial cell adherens junctions is dominant over that of VEGF. Reiss and colleagues reported that the unsaturated free fatty acids increase membrane fluidization of endothelial cells therefore increasing the ADAM10-dependent VE-cadherin proteolysis [42]. Since ANGPTL4 inhibits the LPL, therefore reducing fatty acids release, it is speculated that restriction of the fluidization process might occur using this mechanism.

Previous study reported that P-Y1175-VEGFR2 is strongly involved in VEGF-induced migration [43]. Present data suggest that regulation of endothelial cell migration and fluidization by ANGPTL4 might involve modulation of the P-Y1175-VEGFR2/ERK1-2 pathway. ERK1-2 activation would regulate unjamming and fluidization process therefore inducing collective migration, as reported in tumor cells [44]. We here show that the P-Y1175-VEGFR2 is induced at 2-min and is also strongly sustained after 5-h VEGF treatment. Whereas ANGPTL4 alone does not stimulate VEGFR2 phosphorylation, it further increases the short- and long-term effect of VEGF on P-Y1175-VEGFR2 that translates into distinct kinetics of ERK activity. VEGF-induced short-term ERK1-2 phosphorylation is decreased by ANGPTL4 whereas the long-term is increased. These data suggest that the short-term regulatory effect of ANGPTL4, i.e., decreased cell elongation and migratory phenotype could be mediated by the decreased ERK1-2 phosphorylation. As for the long-term regulation, it has been reported that Src associated to the phosphorylated VEGFR2 mediates the binding of Shb to the P-Y1175-VEGFR2, which is required for VEGF-induced migration of endothelial cells [43]. We previously reported that the ANGPTL4/α_v_β_3_ integrin interaction leads to Src sequestration away from VEGFR2 and maintains the VEGFR2–VE-cadherin at the endothelial surface [16]. The long-term regulatory effect of ANGPTL4 could therefore be mediated by the Src signaling pathway involved in both preservation of the VE-cadherin containing junctions and reduction of the migratory capacities.

This study further confirms that the dynamic ratio of hypoxia-induced proteins which are highly variable in a pathological context leads to a complex regulation of permeability and angiogenesis. Since ANGPTL4 and VEGF are secreted proteins that bind to the ECM through heparan sulfates proteoglycans [24], this work therefore suggests that the narrow environment of the endothelial sprout participates in the fine tuning of capillary morphogenesis by affecting bioavailability of VEGF and ANGPTL4 that thus supports the context-dependent role of ANGPTL4.

## Data Availability

The data presented in this study are available on request from the corresponding author. The data are not publicly available at this time due to technical or time limitations.

## Supplementary Materials

The following are available online at https://www.mdpi.com/2227-9059/10/2/206/s1. Figure S1: Morphometric analysis of the 3D endothelial capillary network sprouting from a bead in fibrin hydrogel. Figure S2: Distribution per length range of capillaries depending on treatment. Figure S3: Endothelial capillaries induced by FLD-ANGPTL4. Figure S4: Protein amount of adherens junction components. Figure S5: Protein amount of VEGFR2 for short- and long-term stimulations. Video S1: Three-dimensional reconstruction of the endothelial capillary network sprouting from a bead in fibrin hydrogel.
