# Supplementary material for: Angiopoietin-like 4-Induced 3D Capillary Morphogenesis Correlates to Stabilization of Endothelial Adherens Junctions and Restriction of VEGF-Induced Sprouting
Source: Biomedicines. 2022 Jan 18;10(2):206. doi: 10.3390/biomedicines10020206 (PMC8869696; doi:10.3390/biomedicines10020206)
Supplement: Supplementary file 1 [file biomedicines-10-00206-s001.zip › Liabotis, Supplementary File Final.pdf]

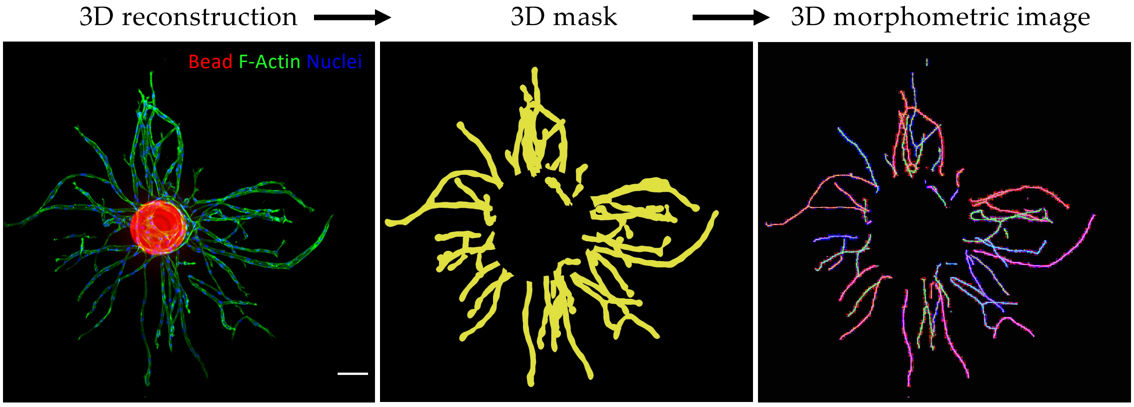


**Supplementary Figure S1.** Morphometric analysis of the 3D endothelial capillary network sprouting from a bead in fibrin hydrogel. Sequential analysis of the 3D images (hereby recapitulated in 2D views). From initial 3D reconstruction from Z-stack images (left panel), a mask is generated after applying a sphere centred on the bead centre and whose radius is 25 µm-increased from the bead radius and thresholding the F-actin signal (middle panel). 3D morphometric image of capillaries is generated from the 3D mask (right panel). Each capillary is a single continuous structure starting from the sphere defined in the 3D mask and finishing at one or several end-points (depending on the branching process). Capillaries are identified individually using single-colour assignment. Several parameters for each bead are quantified from 3D morphometric image, number of end-points (voxel with less than two neighbours), number of branch-points (voxel with more than two neighbours), number of capillaries, total length of capillaries and capillary diameter. Scale bar : 100 µm.

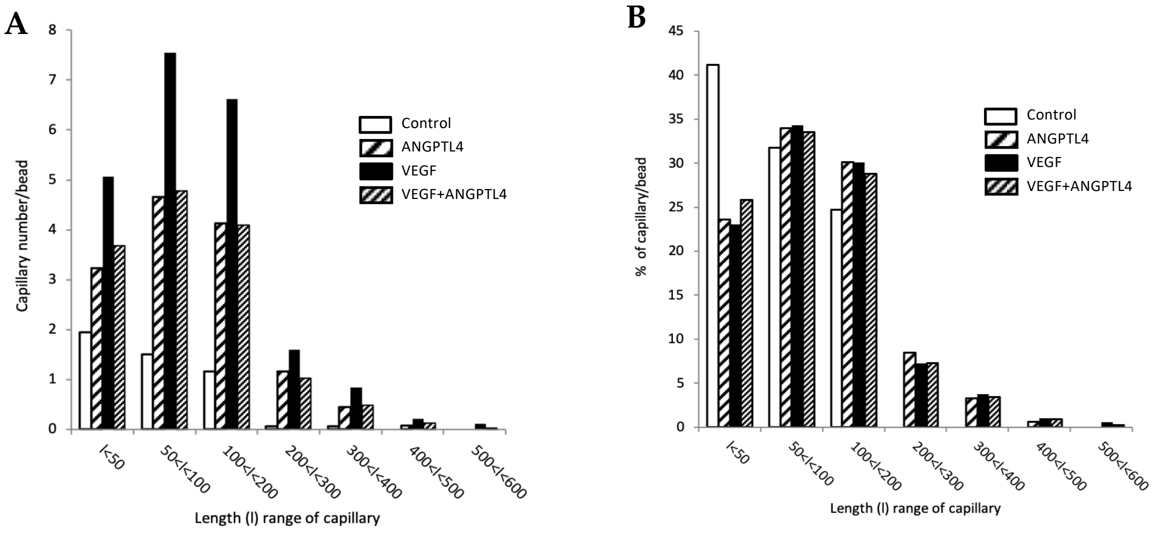


**Supplementary Figure S2.** Distribution per length range of capillaries depending on treatment. 3D capillary formation was assessed after 4 days of culture in absence (control) or presence of ANGPTL4 (2.5 µg/mL) and/or VEGF (2.5 ng/mL). The capillary length (l in µm) is the sum of the length of the main and the related branches. (**A**) Distribution per length range of the mean of capillary number per bead. (**B**) Distribution of percentage per length range of capillaries over the total number obtained in each condition.

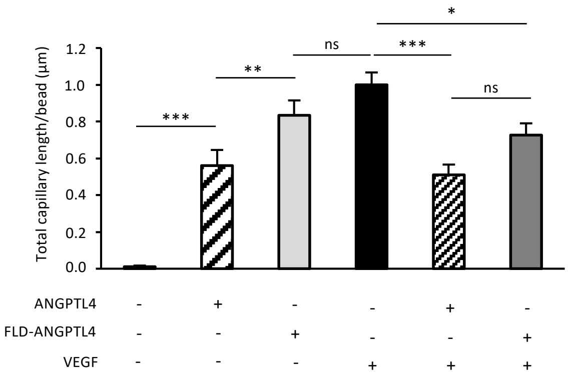


**Supplementary Figure S3.** Endothelial capillaries induced by FLD-ANGPTL4. 3D capillary formation was assessed after 4 days of culture in absence or presence of ANGPTL4 (2.5 µg/mL) or FLD-ANGPTL4 (4 µg/mL) and/or VEGF (2.5 ng/mL). Individual values of total capillary length/bead are normalized to the mean value of VEGF condition in each experiment. Means of the normalized values are then determined (+SEM). Values were measured in 2 independent experiments of quadriplicate wells. ns: *P*>0.05, **P*≤0.05, ***P*≤0.01, *** *P*≤0.001 (one-way ANOVA).

**A**

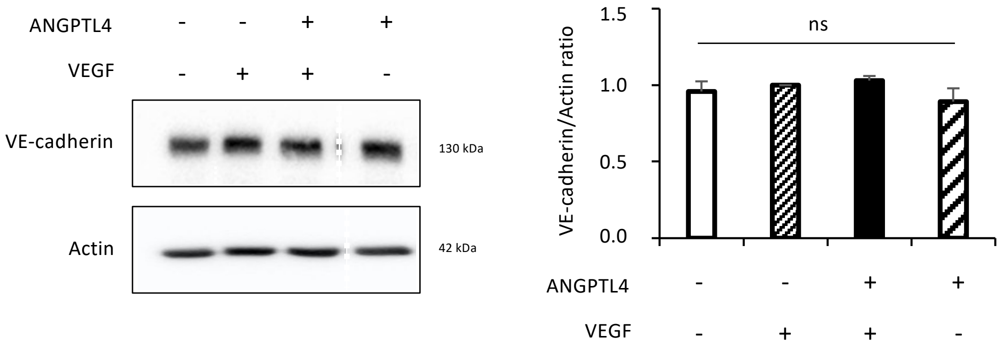


**B**

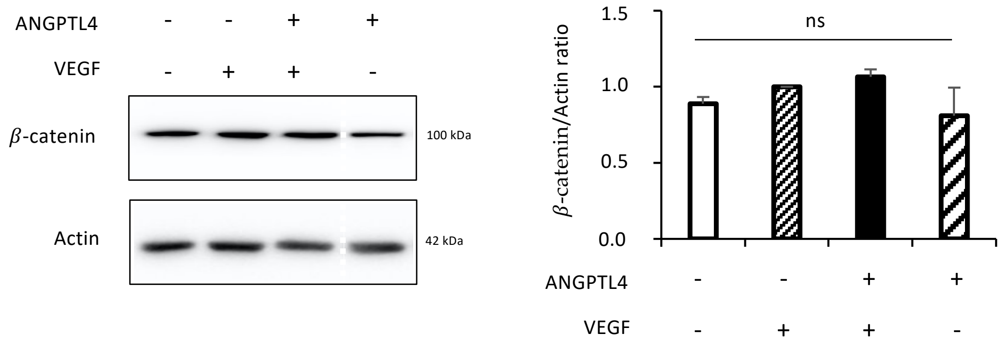


**Supplementary Figure S4.** Protein amount of adherens junction components. Endothelial cells were stimulated in absence or presence of ANGPTL4 (2.5 µg/mL) and/or VEGF (50 ng/mL). Total amount of VE-cadherin (**A**) and $\beta$-catenin (**B**) were analyzed after 5 hours stimulation. Immunoblots are representative of several independent experiments (5 for A and 4 for B). Blot image cuts are indicated by dotted white lines. Corresponding actin immunoblot was used for each VE-cadherin or $\beta$-catenin blot. Graphs represent mean values + SEM of the ratio of VE-cadherin or $\beta$-catenin over actin. Individual values are normalized to the mean value of VEGF condition for each experiment. ns: $P > 0.05$ (one-way ANOVA).

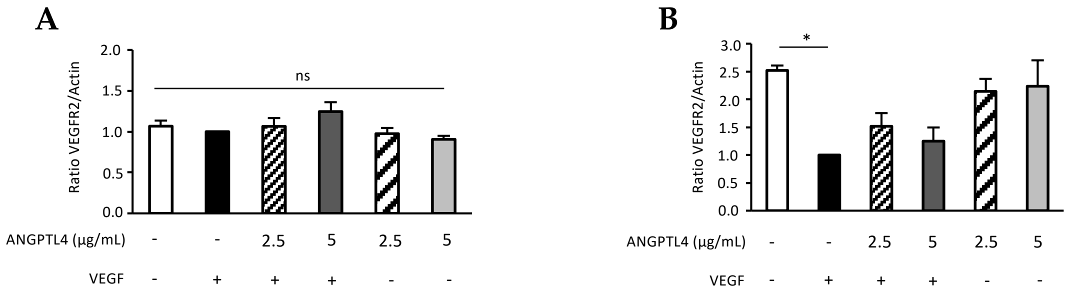


**Supplementary Figure S5.** Protein amount of VEGFR2 for short- and long-term stimulations. Endothelial cells were stimulated in absence or presence of ANGPTL4 (2,5 µg/mL or 5 µg/mL) and/or VEGF (50 ng/mL) for 2 minutes (**A**) or 5 hours (**B**). Total amount of VEGFR2 was analyzed by immunoblot. Graphs represent mean values (+SEM) of the ratio of VEGFR2 over actin. Individual values are normalized to the mean value of VEGF condition for each experiment. ns: P > 0.05, *P≤0.05 (one-way ANOVA).

**Video S1.** Three-dimensional reconstruction of the endothelial capillary network sprouting from a bead in fibrin hydrogel. 3D reconstruction is performed from the Z-stack images acquired in three channels (DsRed, EGFP and DAPI). Capillaries sprouting from the fluorescent bead (red) are stained for F-actin by phalloidin (green) and for nuclei by DAPI (blue). Scale bar : 200 μm.
